# Experimental and Theoretical Analysis of Mechanical Properties of Graphite/Polyethylene Terephthalate Nanocomposites

**DOI:** 10.3390/polym14091718

**Published:** 2022-04-22

**Authors:** Basheer A. Alshammari, Mokarram Hossain, Asma M. Alenad, Abdullah G. Alharbi, Bandar M. AlOtaibi

**Affiliations:** 1 Material Research Institute, King Abdulaziz City for Science and Technology, Riyadh 12354, Saudi Arabia; 2 Zienkiewicz Centre for Computational Engineering (ZCCE), Faculty of Science and Engineering, Swansea University, Swansea SA1 8EN, UK; mokarram.hossain@swansea.ac.uk; 3 Chemistry Department, College of Science, Jouf University, Sakaka 75471, Saudi Arabia; amenad@ju.edu.sa; 4 Electrical Engineering Department, Faculty of Engineering, Jouf University, Sakaka 42421, Saudi Arabia; agalharbi@ju.edu.sa; 5 The National Center for Energy Storage Technologies, King Abdulaziz City for Science and Technology, Riyadh 11442, Saudi Arabia; bmalotaibi@kacst.edu.sa

**Keywords:** graphite nanoplatelets (GNP), poly (ethylene terephthalate) (PET), nanocomposites, mechanical tests, mathematical models

## Abstract

In this work, graphite nanoplatelets (GNP) were incorporated into poly (ethylene terephthalate) (PET) matrix to prepare PET-GNP nanocomposites using a melt compounding followed by compression moulding and then quenching process. Both static and dynamic mechanical properties of these quenched materials were characterized as a function of GNP contents using dynamic mechanical thermal analysis (DMTA) and tensile machine, respectively. The results demonstrated that the addition of GNP improved the stiffness of PET significantly. Additionally, the maximum increase in the storage modulus of 72% at 6 wt.% GNP. The incorporation of GNP beyond 6 wt.% into PET decreases the storage moduli, but they remain higher than pure PET. The observed reduction could be due to agglomeration, resulting in poorer dispersion and distribution of higher levels of GNP into the PET matrix. In contrast to the results for moduli, tensile strength and elongations at break reduce with increasing the GNP content. For example, tensile strength reduced from ∼46 MPa (neat PET) to ∼39 MPa (−15%) for the nanocomposites containing 2 wt.% GNP. This reduction is accompanied by a decline in elongation at break from ∼6.3 (neat PET) to ∼3.4 (−46%) for the same nanocomposites. Such reductions are followed by a gradual decrease in upon further addition of GNP. These reductions indicate that increasing GNP loadings, results in brittleness in nanocomposites. In addition, it was found that quenched PET and composite samples were not fully crystallized after processing and therefore (cold) crystallized during the first heating cycle DMTA, as indicated by a rise in storage moduli above the glass transition temperature during the DMTA first heat. Furthermore, mathematical models based on non-linear theories are developed to capture the experimental data. For this, a set of mechanical stress-strain data is used for model parameters’ identification. Another set of data is used for the model validation that demonstrates good agreements with the experimental study.

## 1. Introduction

Carbon has an extraordinary ability to join within itself and with various other elements. The most frequently used and widely investigated particulate carbon fillers include graphite, carbon black, graphene, graphite nanoplatelets, carbon fibre and carbon nanotubes that are used to fabricate polymer matrix composites for an extensive variety of applications. These applications include various parts in aerospace industries, productions of tidal and wind turbine blades for harvesting renewable energy, various components in automobile industries etc. [1,2]. Conventionally, micro-size fillers are used to improve the properties of polymer matrix. However, nano-size fillers have been utilized instead of the micro-fillers as these nano-fillers offer marked improvement to the properties of polymers due to their high aspect ratio compared to the micro-filler counterparts. Nanocomposites have a huge interfacial area between polymers and fillers. Such huge area generates a significant volume fraction of polymer in close contact to the interface/interphase with properties that vary from the bulk properties of the polymer even at low level of nano-fillers. Previous studies revealed that low contents of nanoscale fillers could cause remarkable changes in thermal, electrical, and mechanical properties of polymers and their composites. These enhancements are mostly related to improvements in crystallinity, strength, conductivity, and thermal stability. The most important advantages of nano-fillers over micro-fillers are the opportunity of multifunctional properties and the decrease of the quantity of filler required to achieve desirable properties [3,4,5,6,7]. Several processing techniques for dispersion and incorporating these nano-fillers into a polymer matrix have been investigated including in-situ polymerization, solution and melt compounding. The latter method is one of the most common techniques due to its mass production of composites containing thermoplastic polymers. In the melt compounding method, there is no solvent as well as traditional equipments such as extruder and internal mixer for polymer processing can be utilized for the composites [6,8,9].

Graphene is a well-known strong and stable material for improving both mechanical and thermal properties of a polymer matrix. However, many challenges obstruct their extensive use for polymer composite applications. These challenges include high manufacturing cost, difficult of processing and homogeneity of its dispersion into the polymer matrices [10,11]. Consequently, graphene nanoplatelet (GNP) has a great potential as a nano-filler for polymer matrices. GNP has many layers of graphene (up to 60) and is usually prepared by the modification of graphite materials [8] which allow certain atoms, molecules and ions to be inserted between its graphene sheets. Usually natural graphite is immersed in a concentrated sulphuric acid solution with other oxidizers such as ozone, nitric acid, hydrogen peroxide, potassium permanganate. The resulting products are known as graphite intercalation compounds (GIC) and also as expandable graphite. The GIC is heated to a temperature of about 1000 °C for 15–30 s which generates large amounts of gases such as SO_2_ and H_2_O resulting in further expansion of the graphite; which is known as exfoliated or expanded graphite (EG). Further exfoliation of EG can produce thin layers of graphite to obtain the GNP in 2D form. The degree of dispersion and properties of final polymer composites depend on several factors including the number of graphene layers, type of polymers, filler loading, fabrication method, dispersion state, and adhesion (interfacial interaction) between the fillers and the matrix [12,13,14,15].

Poly (ethylene terephthalate) (PET) is an aromatic polyester which is a common thermoplastic polymer used in applications such as packaging due to its excellent properties such as clarity, wear-resistance, hardness, chemical resistance, thermal, and dimensional stability etc. [16,17,18,19]. Moreover, PET has a high glass transition temperature ($T_{g}$) of around 85 °C and melting point of about 255 °C. Yang et al. [20] dispersed GNP into recycled PET, which caused an obvious improvement in storage modulus and its viscosity. Xu et al. [21] has carried out another study by preparing PET-GNP composites and observed an enhancement in crystallization rate and thermal stability. Similar results have reported by Chowreddy et al. [22] for PET composites containing carbon nanotubes (CNTs). Xie and co-workers [23] fabricated PET composites containing CNTs and reported that CNTs acted as nucleating agents and significantly accelerate the crystallization rate. Similar composite has prepared by Tasic et al. [24] who stated that mechanical properties of composites improved upon the incorporation of CNTs. Zhang and workers [25] prepared PET composites containing graphene with the help of a melt compounding method. They concluded that a continuous network path has been observed which caused an improvement in their electrical conductivity. Mallakpour et al. [26] found electrical conductivity of the PET composites containing 0.1 wt.% of CNTs increased 10 times of the magnitude. This attributed to formation of a continuous conductive path. Nanoclay has been incorporated into PET by Barbosa et al. [27] who found that nanoclay acted as nucleating agents and significantly enhanced the crystallization rate. Inuwa et al. [28] prepared hybrid PET composites containing both GNP and CNTs. They noticed that the mechanical properties were improved with the incorporation of GNP and CNTs into the PET matrix. However, the enhancement in these properties was higher in the case of GNP compared with CNTs. Mansor et al. [29] reviewed the fundamental aspects of morphology and surface group of nanocomposites containing CNTs and graphene.

Although the above-mentioned studies have investigated the properties of PET-GNP nanocomposites, to the best of authors’ knowledge, there exists no study that has investigated mechanical properties such as stress-strain curves, tensile strength, elongation at break etc., of PET-GNP nanocomposites both experimentally and theoretically. In this work, nanocomposites materials were fabricated using PET as the matrix containing GNP as nano-fillers with amount ranging from 2 to 10 wt.%. Then these nanocomposites were investigated through both static and dynamic mechanical properties including tensile strength ($\sigma_{u}$), tensile modulus (E), elongation at break ($\epsilon_{u}$), storage modulus (E${}^{\prime }$), loss modulus (E″), tanδ and glass transition temperature ($T_{g}$) under controlled cyclic strain of the composites as a function of GNP contents using a dynamic temperature analysis (DMTA) and a uniaxial tensile test machine. One of the key contributions of this current paper is it develops a mathematical framework at large strains for capturing the uniaxial tensile data of PET-GNP nanocomposites performed here. Once the model is calibrated using one set of experimental data, it is further validated with another set of fresh data that was not used in the parameter identification.

## 2. Experimental Work

### 2.1. Materials

PET polymer received as resin and supplied from Equi-polymers company (LIGHTER C93, Schkopau, Germany). It has a glass transition temperature of 74 °C, melting temperature ∼245 °C and a density of 0.89 g/cm^3^. GNP (GNP grade xGnPs-15 used in this study) was purchased from XG Sciences (Lansing, MI, USA). Its average diameter and thickness are ∼15 μm and 6–8 nm, respectively, and is composed of short stacks of graphene sheets as per standard specifications by the supplier.

### 2.2. Preparation of PET-GNP Composites

At first, both matrix and carbon fillers were dried at about 120 °C in vacuum oven overnight before any processing and characterization were carried out. The preparation of the PET/carbon composites was carried out using a laboratory scale extruder (Thermo-Haake Minilab, capacity: 7 cm^3^) equipped with co-rotating twin-screws. The mixing time, temperature, and screw speed were set at 5 min, 280 °C, and 45 rpm, respectively. We have tried with other parameters. However, these are optimized as these seem to be good to process. The extruded samples were compression molded at 280 °C for 10 min and quenched in an ice bath for 30 s as to reduce the crystallinity and enhance ductility for easy sampling. Then, the compression moulded samples were chopped, dried at 40 °C for 24 h and stored for the dynamic mechanical analysis and characterizations.

### 2.3. Characterization of PET-GNP Composites

**Uniaxial tensile tests**: Specimens for tensile testing were prepared using an injection moulding machine. The tensile testing was performed using an Instron instrument of model 4301 at a crosshead speed of 5 mm/min by following ASTM D638 standard. The average of five test results was reported as a final value of tensile properties. All the tensile tests were carried out at room temperature.

**Dynamic Mechanical Thermal Analysis (DMTA)**: DMTA was conducted using a DMA Q-800 machine (TA Instruments) in a single cantilever mode. The specimen of dimensions 40 × 10 × 1 mm^3^ were heated from 23 °C to 220 °C at the heating rate 3 °C/min. The sample was deformed at a constant amplitude of 10 μm and a frequency of 1 Hz. The curves of storage modulus (E${}^{\prime }$), loss modulus (E″), and tanδ were obtained as a function of temperature via Universal Analysis Software that is installed within the DMTA system.

## 3. Results and Discussions

**Figure 1:**
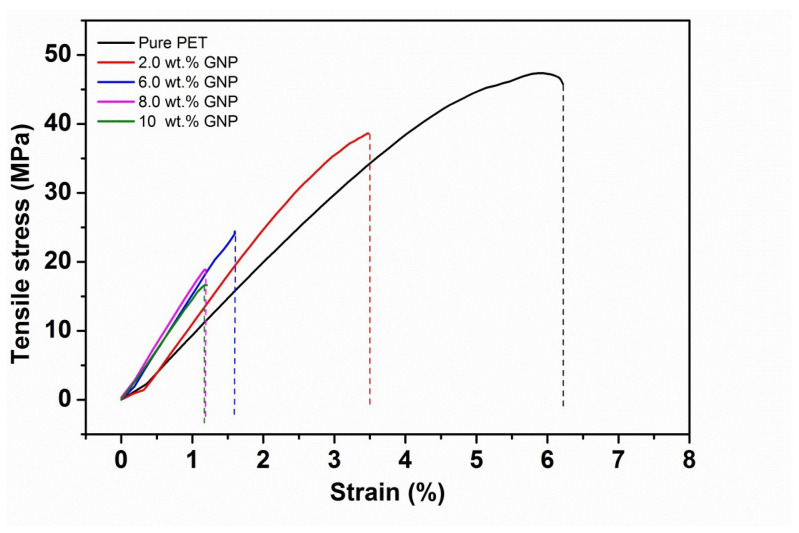
Typical stress-strain curves of the PET-GNP nanocomposites.

**Figure 2:**
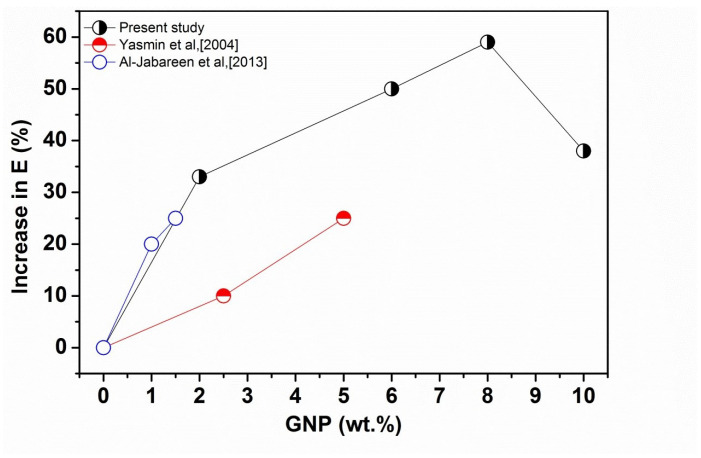
Comparison of the elastic moduli data from Yasmin et al. [31] (Epoxy matrix) and Al-Jabareen et al. [32] (PET matrix) with the present experimental results.

Figure 1 shows the stress-strain curve of PET-GNP nanocomposites with different GNP loadings and the data obtained from these curves is shown in Table 1. The results show that the elastic modulus (E) improves with increasing GNP loadings up to 8 wt.%. The elastic modulus of pure PET is 1073.3 MPa which increases to ∼1709 MPa at 8 wt.% GNP (+59%). This is followed by reduction at 10 wt.% of GNP. However, E values are still higher than pure PET, indicating a greater degree of reinforcement and/or inducement of a higher degree of crystallinity in the case of nanocomposites. Furthermore, as per extrapolation results, the E values were improved by ∼11% due to the increase in crystallinity ($X_{c}$) and by 48% by the reinforcement effect of GNP. The degree of crystallinity of these nanocomposites were shown in our previous work [30]. Extrapolation of the modulus data (up to 2 wt.%) shown in Table 1 to 100% GNP gives a value of ∼18.9 GPa as an estimate of the effective modulus of the GNP in these nanocomposites which is much higher than that of graphite (∼6 GPa) in the microcomposites, indicating a greater reinforcement effect of the nano-filler over the micro-filler. Several studies have been reported the improvement in elastic modulus values for polymer/GNP nanocomposites such as Yasmin et al. [31] and Al-Jabareen et al. [32]. Figure 2 shows comparative tensile moduli as a function of GNP content of polymer/GNP nanocomposites obtained from these studies compared with the present experimental data. It can be clearly seen in figure that as the GNP content is increased, the values of elastic moduli is increased for all composites. To ensure accuracy and reproducibility, five tests were performed and the average of them was reported as the final value of tensile properties.

### 3.1. Tensile Properties of PET-GNP Nanocomposites

However, data from the present study is equivalent to or greater than data from the other studies up to 8 wt.% of GNP, followed by a reduction in the values. The data obtained from the present study and Al-Jabareen et al. (PET-GNP) are close to each other in comparison to Yasmin et al. (Epoxy/GNP), reflecting the similarity in materials and methods for making composites. In contrast to the results for the elastic moduli, Figure 1 and Table 1 demonstrate that $\sigma_{u}$ and $\epsilon_{u}$ reduce with the increase of GNP content. For example, $\sigma_{u}$ reduced from ∼46 MPa (neat PET) to ∼39 MPa (−15%) for the nanocomposites containing 2 wt.% GNP. This reduction is accompanied by a decline in $\epsilon_{u}$ from ∼6.3 (neat PET) to ∼3.4 (−46%) for the same nanocomposites. Such reductions are followed by gradual decreases in $\sigma_{u}$ and $\epsilon_{u}$ upon further additions of GNP. These reductions indicate that increasing the GNP loadings result in brittleness in nanocomposites, as discussed for microcomposites in the previous section.

**Figure 3:**
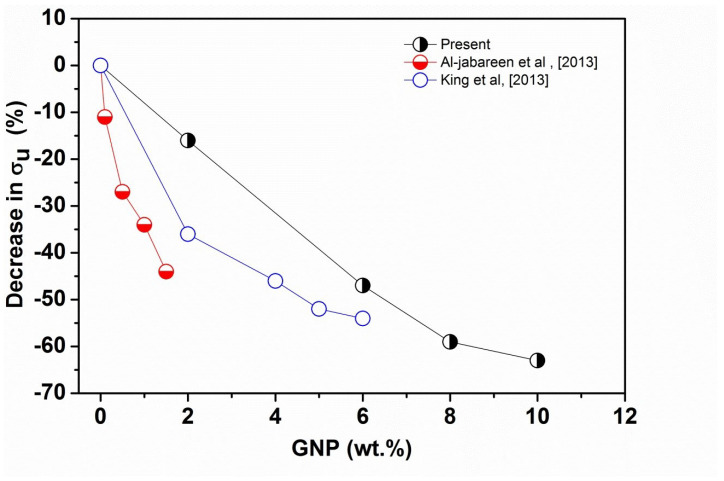
A comparison of $\sigma_{u}$ data from Al-Jabareen et al. [32] (PET matrix) and King et al. [33] (Epoxy matrix) with the present experimental results.

Moreover, similar results have been reported for PET-GNP nanocomposites by Al-Jabareen et al. [32] who observed that the addition of 1.5 wt.% of GNP in the PET matrix results in a decline in $\sigma_{u}$ and $\epsilon_{u}$ values by 45% and 60%, respectively. Moreover, King et al. [33] studied the effects of GNP addition on the tensile properties of epoxy and reported an increase in the tensile modulus but a reduction by −54% and −81% in the values of $\sigma_{u}$ and $\epsilon_{u}$, respectively. Yasmin et al. [31] reported brittleness of composites containing more than 5 wt.% loading of graphite, which was accompanied by a decline in the $\sigma_{u}$ and $\epsilon_{u}$. In addition, an agglomeration of graphite was observed leading to a reduction of these properties for epoxy/graphite composites. Figure 3 shows $\sigma_{u}$ as a function of GNP content for polymer/GNP nanocomposites obtained from references [32,33] compared to the present experimental data. It is clear that as GNP content is increased, $\sigma_{u}$ decreases. However, the reduction in the present study is much lower compared to the two previous studies up to 6 wt.% of GNP. This could be due to the thickness and length of GNP as well as GNP loadings, For example, Jabareen et al. [32] used thickness and diameter of the GNPs were estimated to be 4.5 nm and 46 μm, respectively and up to 1.5 wt.% of GNP. While in the current study, we used GNPs having 7 nm and 15 μm, respectively, and up to 10 wt.% of GNP.

### 3.2. DMTA of PET-GNP Nanocomposites

**Figure 4:**
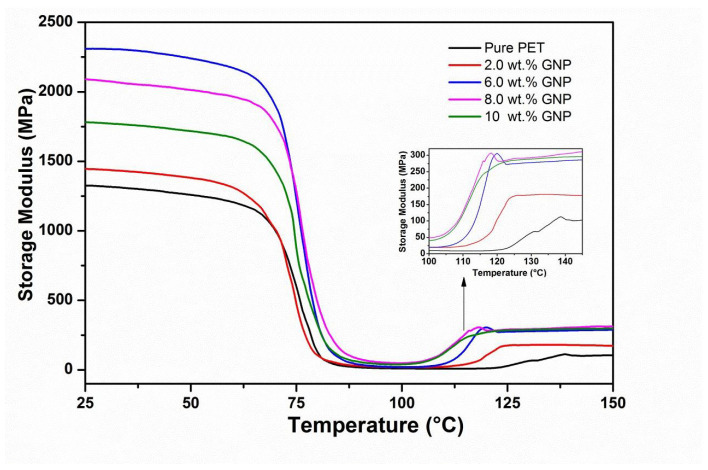
DMTA dynamic storage modulus data of PET-GNP nanocomposites.

The storage modulus (E${}^{\prime }$) versus temperature curves for the PET matrix and nanocomposites with different loadings of GNP (2, 6, 8, 10 wt.%) are presented in Figure 4, and Table 2 shows comparative E${}^{\prime }$ values at approximately room temperature (25 °C) and above $T_{g}$ (100 °C). Figure 4 shows curves of similar characteristics to those obtained for the PET/graphite composites [33], the E${}^{\prime }$ curves for all the samples are essentially the same shape; i.e., values of E${}^{\prime }$ (although different for each material, (see Table 2). All results show relatively little reduction in the glassy region below $T_{g}$ (∼80 °C) and then decrease dramatically following the glass transition region reaching minimum values at ∼100 °C. However, in contrast to the graphite micro-composites, the values of E${}^{\prime }$ are generally somewhat higher, indicating a greater degree of reinforcement and/or inducement of a greater degree of crystallinity [34]. Furthermore, upon increasing the temperature above 100 °C, an increase in the values of E${}^{\prime }$ for all nanocomposites samples is observed as shown in the insert of Figure 4. This is due to cold crystallization of the amorphous regions developed during the quenching process. It is also obvious that the cold crystallization of the GNP nanocomposites begins at lower temperatures than for the pure PET (Figure 4).

In addition, the increase in moduli following cold crystallization is much greater in Figure 4 and Table 2. This behavior indicates that GNP nucleates the cold crystallization of the PET matrix more efficiently, which may be due to their greater surface area. Generally, nucleation represents the initial step of crystallization that can be explained as the development of region of short-range order in the polymer, which act as growth centers for crystallization. It is worth mentioning the phenomenon called cold crystallization, which has been observed experimentally after quenching the polymer from above its melting point to below its glass transition temperature to form a randomly oriented glass as the crystallization is inhibited below this temperature. Table 2 shows the maximum increase in E${}^{\prime }$ of 72% at 6 wt.% GNP. The incorporation of GNP beyond 6 wt.% into PET decreases the E${}^{\prime }$ values, but they remain higher than pure PET. The observed reduction could be due to the agglomeration, resulting in poorer dispersion and distribution of higher levels of GNP into the PET matrix as reported in our previous work [30] for same samples. In addition, there is the possibility of GNP to rolling up during melt blending as mentioned earlier, which reduces both their aspect ratio and interfacial area. In fact, the value of E${}^{\prime }$ at 10 wt.% is very close to the value for graphite at the same wt.%, indicating that the effective modulus of GNP has reduced to that of graphite [34]. Note that the glass transition temperature of polymers was found to be dependent on the free volume that is available for the movement of polymer chains. Thus, GNP content may have influence on the free volume that is indicative of slight improvement in $T_{g}$ of composites.

**Figure 5:**
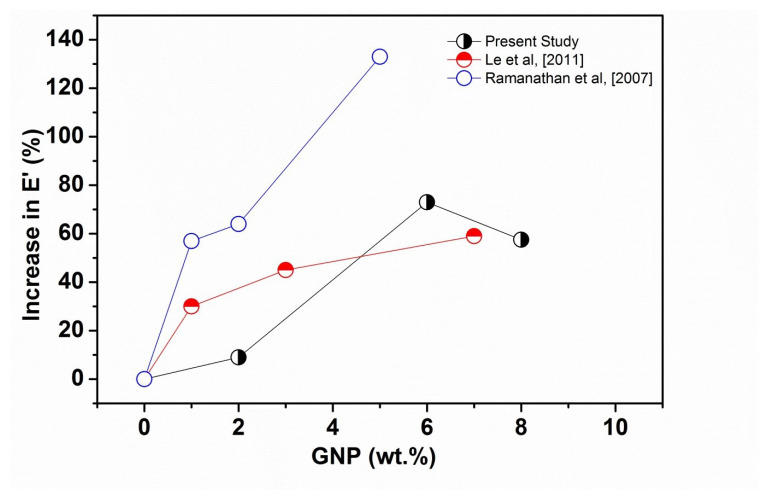
Comparison of E${}^{\prime }$ data from reference [35] (PMMA matrix) and [30] (PET matrix) with the present experimental results.

Figure 5 shows comparative storage moduli as a function of GNP content of polymer/GNP nanocomposites obtained from the literature [35,36] compared with the present experimental data. The highest E${}^{\prime }$ values can be clearly seen in this figure for the data taken from Ramanathan et al. [35]; which could be due to the solution preparation method that was used to produce the GNP nanocomposites in their study. Melt compounding was utilized in Li et al. [36] and their reported results are much closer to the present study. The same authors also studied the effects of GNP on the dynamic mechanical properties of Poly(trimethylene terephthalate) (PTT) and poly(butylene terephthalate) (PBT) matrices; reporting enhancement in E${}^{\prime }$ (at ∼25 °C) of 112% and 66% for PTT [37] and PBT [38] nanocomposites, respectively, at 7 wt.% GNP. Such improvement was attributed to an efficient load transfer from matrix to filler, resulting from a uniform distribution and good interfacial adhesion between GNP and the thermoplastic matrices. Some authors [35,36,37,38,39,40] have compared the effect of graphite and GNP on the E${}^{\prime }$ values and generally observed that the addition of GNP gave greater improvement in the E${}^{\prime }$ values than graphite. This is attributed to the small size particles, high aspect ratio, and large surface area GNP compared to graphite. Those results seem to be in good agreement with the results in the current study.

**Figure 6:**
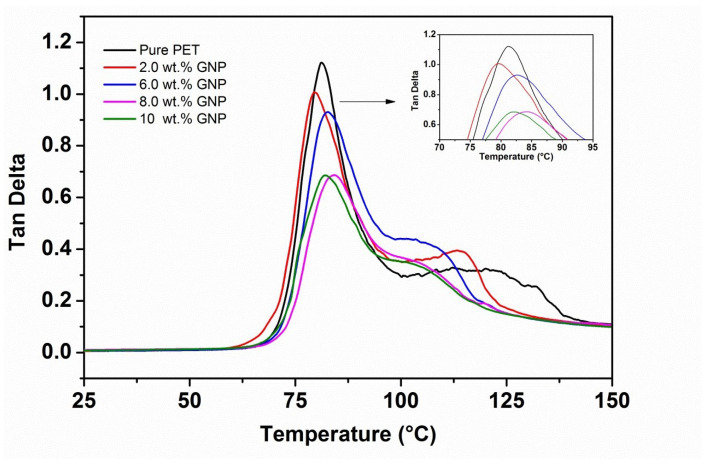
DMTA tanδ versus temperature data for the PET-GNP nanocomposites.

Figure 6 shows tanδ versus temperature for PET-GNP nanocomposites system and Table 2 summarises values of $T_{g}$ and tanδ obtained from this figure. It is clear that the intensity of the tanδ peaks decline for the nanocomposites compared to pure PET. The tanδ values reduced from ∼1.15 to 0.69 for pure PET to PET-GNP nanocomposites containing 8 wt.% GNP. The reduction in tanδ values could be attributed to both an increase in the degree of crystallinity from 11.8% (pure PET) to 22% for the PET nanocomposites. It has been reported that the area under the tanδ peaks, usually decreases with increasing GNP [36], which is attributed to the two-dimensional (2D) structure of the graphene sheets which hinders the transition from the rigid glassy to the rubbery state. The DMTA results of the present study agree with previous studies [41] in terms of enhancement of E${}^{\prime }$ and reduction of tanδ values upon incorporating GNP within polymer matrices. However, the $T_{g}$ values of PET-GNP nanocomposites indicate no appreciable change (Table 2). Similar observations of $T_{g}$ and tanδ have been reported for different matrices such as PET-GNP [36], PTT-GNP [37] and PBT-GNP [38] nanocomposites. Li et al. [36] reported a slight increase in $T_{g}$ values and attributed this behaviour to higher filler surface area and the possibility of the existence of functional groups which can promote the attraction of chain segments of PET onto the GNP surface thereby restricting their mobility.

## 4. Constitutive Modelling of Filled Polymers

In this section, we will concentrate on the development of appropriate mathematical models to capture the mechanical behaviour of filler-reinforced polymers performed in this study. Mathematical modelling of particle-filled polymers has been an active field of research for the last several decades [42,43,44]. In contrast to some classical models that are mainly based on linear strain theories, a wide range of constitutive models has been proposed over the years to capture large strain behaviours of particle-filled polymeric materials [34,44]. In the following sections, both types of models will briefly be discussed.

### 4.1. Small Strain-Based Particle-Filled Polymer Models

**Figure 7:**
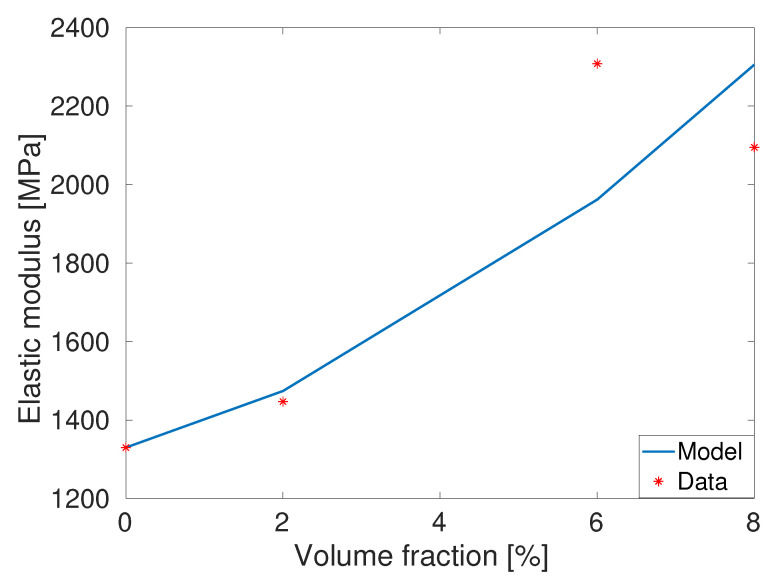
Model fitting of the elastic moduli at various fraction of PET-GNP fillers.

In the case of mathematical models for particle-filled polymers using small strain Hookean theory, one of the earliest and simplest approaches was due to Guth [42], Guth and Gold [43] in which the prediction of one of the most important linear elastic constants, i.e., Young’s modulus of a particle-filled solid is expressed as $E_{c}=E_{m}\left(1+2.5v_{f}\right)$ where $E_{c},E_{m}$ are the Young’s moduli of the composite (*c*) and the matrix (*m*), respectively and $v_{f}$ is the weight fraction of filler (expressed mainly in percentage). According to the small strain (Hookean) theory, this estimate however is only good at very low filler concentrations and small deformation ranges (<1%). Therefore, a number of attempts have been made to improve model predictions by adding more terms to a polynomial series expansion of the amplification factor. One of the most widely-used forms is as follows

(1) $$\begin{array}{c} E_{c}=E_{m}\left[1+0.67gv_{f}+1.62{\left[gv_{f}\right]}^{2}\right], \end{array}$$

where the shape factor *g* is related to length/breadth of a particle cluster and its value is assumed larger than one (unity). There are other small-strain based material models for composites that can easily be incorporated. In order to identify the material constant *g* appearing in Equation (1), we used the experimental data for the PET-GNT nanocomposites presented in Table 2. After using the four set of experimental data at various percentages of fillers with the help of a simultaneous optimisation routine from Matlab, the parameter *g* is identified as g=6.21 and the corresponding fitting result is presented in Figure 7.

### 4.2. Large Strain-Based Particle-Filled Polymer Models

The mechanical tests conducted on PET-GNP nanocomposites clearly demonstrated that the particle-filled polymers are deformable far beyond than one percent (>1%) deformations. Hence, mathematical models that are suitable for capturing the strain-stress data at large strains must be utilised. Note that these finite strain-based models have been developed mainly around the concept of the amplification factor (*X*) in a homogenised sense. For modelling polymeric materials at large deformations, the starting point is the deformation gradient F that represents large strain deformations where the left Cauchy-Green strain tensor is defined as $b=FF^{T}$. In general, polymeric materials are considered as incompressible solids which can be characterised by an strain energy function as

(2) $$\begin{array}{c} \Psi =\tilde{\Psi }\left(I_{1},I_{2},I_{3}\right). \end{array}$$

In Equation (2), the three strain invariants $I_{1},I_{2},I_{3}$ are defined as $I_{1}=\lambda_{1}^{2}+\lambda_{2}^{2}+\lambda_{3}^{2};I_{2}=\lambda_{1}^{2}\lambda_{2}^{2}+\lambda_{2}^{2}\lambda_{3}^{2}+\lambda_{1}^{2}\lambda_{3}^{2};I_{3}=\lambda_{1}^{2}\lambda_{2}^{2}\lambda_{3}^{2}$, where $\lambda_{1},\lambda_{2},\lambda_{3}$ are the three eigenvalues of the deformation tensor b. The first eigenvalue $\lambda_{1}$ represents the stretch in the *x* direction while $\lambda_{2}$ and $\lambda_{2}$ denote stretches in the *y* and *z* directions, respectively. Note that for the incompressible materials, the third invariant $I_{3}$ becomes unity, i.e., $I_{3}=1$. Henceforth, the Cauchy stress tensor (σ) may be expressed as

(3) $$\begin{array}{c} \sigma =-pI+2\frac{\partial \Psi }{\partial I_{1}}b-2\frac{\partial \Psi }{\partial I_{2}}b^{-1}, \end{array}$$

where *p* is a scalar quantity that serves as an indeterminate Lagrange multiplier which will be removed from the above equation using appropriate boundary conditions. In Equation (3), $b^{-1}$ is the inverse of the deformation tensor b and I is a second order identity tensor. We have conducted all experiments described in the previous sections (Figure 1) that fulfil usual dimensions of a uni-axial deformation where the deformation gradient and the left Cauchy-Green tensor can be defined as in the our previous paper [34].

As it has been demonstrated in our previous publication [34] that in order to predict the behaviour of unfilled polymers, the widely-used classical Carrol model is well-suited. The energy function of Carrol model is as follows

(4) $$\begin{array}{c} \Psi =aI_{1}+bI_{1}^{4}+c\sqrt{I_{2}} \end{array}$$

where a,b,c are material parameters of the Carrol model that need to be identified from appropriate sets of experimental data. Applying the differentiation defined in Equation (3), the uni-axial stress expression for unfilled polymers with Carrol model can be derived as

(5) $$\begin{array}{c} \sigma =\left[2a+8b{\left[2\lambda^{-1}+\lambda^{2}\right]}^{3}+c{\left[1+2\lambda^{3}\right]}^{-\frac{1}{2}}\right]\left[\lambda^{2}-\lambda^{-1}\right]. \end{array}$$

For a detailed derivation of Equation (5), readers may consult the works of Steinmann et al. [45], Hossain et al. [46,47], Shen et al. [48]. To predict the behaviour of filled polymers at large strains, Mullins and Tobin [44] introduced the notion of so-called amplified stretch Λ, which in the case of a uni-axial loading, is related to the actual axial stretch λ by

(6) Λ=1+X(λ−1)

where *X* is the stretch amplification factor that depends on the fraction of filler $v_{f}$. For instance, according to Guth model, this factor can be defined as

(7) $$X=1+0.67gv_{f}+1.62{\left[gv_{f}\right]}^{2}.$$

In order to obtain complete stress-stretch expressions, according to Mullins-Tubin assumption [42,43], the actual stretch λ will be replaced by the amplified stretch Λ

(8) $$\begin{array}{c} \sigma =\left[2a+8b{\left[2\Lambda^{-1}+\Lambda^{2}\right]}^{3}+c{\left[1+2\Lambda^{3}\right]}^{-\frac{1}{2}}\right]\left[\Lambda^{2}-\Lambda^{-1}\right]. \end{array}$$

Note that in our studies, we have calculated the uni-axial engineering stress (or nominal stress that is calculated as the total force over the original sample cross-sectional area) *P* which relates to the uni-axial Cauchy stress via σ=λP.

### 4.3. Parameter Identification and Model Validation

**Figure 8:**
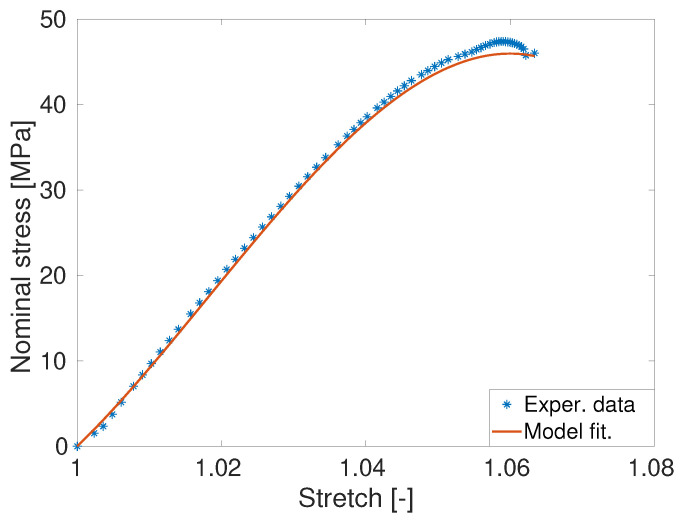
Identification of Carrol model parameters a,b,c using the stress-strain experimental data obtained from the unfilled PET polymer.

**Figure 9:**
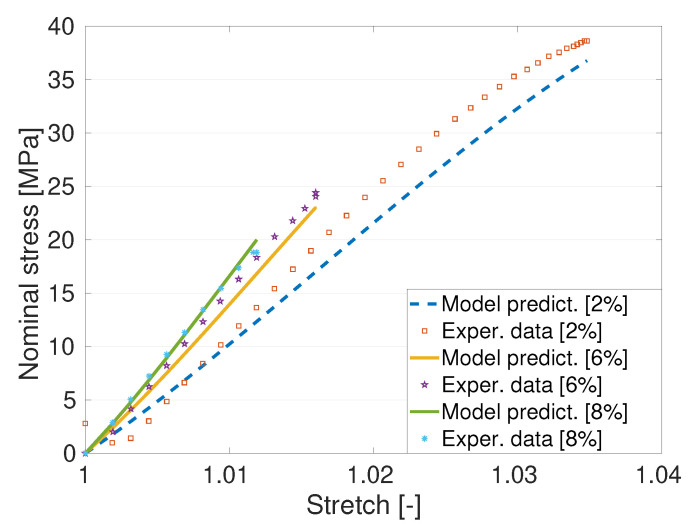
Model prediction using the three-parameter Carrol non-linear model. The predictions are relatively good with the experimental data of three different PET/GNP nanocomnposites.

Carrol model presented in Equation (5) is used to fit the experimental data of the pure (unfilled) PET that breaks at a 6.5% deformation. Figure 8 presents the Carrol model fitting with experimental data of the unfilled PET (asterisks are for the unfilled PET) with the identified parameters as [a,b,c] = [1.44e + 4 MPa, −112.6 MPa, 7493 MPa]. The Carrol model nicely fits the experimental of unfilled PET polymer. Once Carrol model parameter is identified with the help of the unfilled PET data, the model needs to be validated with other data that are not used in the parameter identification process. For the model validation with the filled PET data, the shape parameter *g* appearing in the amplification factor *X* as defined in Equation (6) needs to be identified first. Note that we already identified the amplification factor *g* using the data of the storage moduli from Table 2. In this case, we used the value 6.21 already identified by the linear model of modulus of elasticity enhancements using $E_{c}=E_{m}\left[1+0.67gv_{f}+1.62{\left[gv_{f}\right]}^{2}\right]$. Once all material parameters appearing in Equations (6) and (8) are identified, the model needs to be validated. In this case, predictions of the stress-stretch behaviours of three different PET-GNP materials are presented in Figure 9. All prediction results are in good agreements with the experimental data.

## 5. Conclusions

In this study, graphite nanoplatelets were incorporated into PET matrix using melt-compounding method followed by compression molding assisted with a quenching process. The focus of this study was to investigate the effect of graphite nanoplatelets loading on resultant mechanical and dynamic properties of the nanocomposites. After a series of carefully studies, we found that the storage modulus was increased by 72% at 6 wt.% GNP. Furthermore, tensile strength and elongation at break were obtained by performing mechanical experiments using a tensile test machine. Mechanical experiments depicted that tensile stress was increasing and elongation at break was decreasing, respectively, with the increase of the GNP content. This reduction was followed by gradual decrease in upon further addition of the GNP. The observed reduction could be due to the agglomeration, resulting in poorer dispersion and distribution of higher levels of GNP into the PET matrix. We further developed mathematical models to capture the mechanical (e.g., stress-strain) behaviour that can accurately predict experimental data of unfilled PET as well as filled PET with various percentages of GNP fillers. The study will help in proper selection of GNP fillers in PET or other matrices for specific applications in which mechanical property enhancements are important.

## Figures and Tables

**Table 1:** Tensile properties of the PET-GNP nanocomposites.

GNP (wt.%)	Tensile Modulus (MPa)	Tensile Strength (MPa)	Elongation at Break %
0	1073.3 ± 15.7	45.8 ± 2.3	6.3 ± 1.9
2	1429.5 ± 42.0	38.6 ± 4.7	3.4 ± 0.6
6	1611.3 ± 53.0	24.4 ± 3.4	1.6 ± 0.1
8	1708.7 ± 65.0	18.8 ± 1.3	1.2 ± 0.1
10	1443.2 ± 71.9	16.8 ± 2.5	1.2 ± 0.3

**Table 2:** Selected DMTA data for E${}^{\prime }$, $T_{g}$ and tanδ of PET-GNP nanocomposites.

GNP (wt.%)	E${}^{\prime }$ (MPa) at 25 °C (MPa)	E${}^{\prime }$ (MPa) at 100 °C (MPa)	$T_{g}$ (°C)	tanδ at $T_{g}$
0	1330 ± 92	10.0 ± 2.5	80.5 ± 1.3	1.15 ± 0.10
2	1447 ± 88	22.9 ± 2.2	80.7 ± 0.5	0.93 ± 0.02
6	2308 ± 62	44.6 ± 5.2	82.7 ± 0.5	0.93 ± 0.02
8	2095 ± 84	129.4 ± 14	83.5 ± 0.7	0.69 ± 0.04
10	1782 ± 33	120.0 ± 44	82.1 ± 0.9	0.69 ± 0.10

## Data Availability

Not applicable.
